# Efficacy of a one-catheter concept for transradial coronary angiography

**DOI:** 10.1371/journal.pone.0189899

**Published:** 2018-01-02

**Authors:** Christoph Langer, Julia Riehle, Helge Wuttig, Stephanie Dürrwald, Helmut Lange, Alexander Samol, Norbert Frey, Marcus Wiemer

**Affiliations:** 1 Klinik für Innere Medizin III mit den Schwerpunkten Kardiologie, Angiologie und internistische Intensivmedizin Universitätsklinikum Schleswig-Holstein, Campus Kiel, Christian-Albrechts-Universität Kiel, Kiel, Germany; 2 Kardiologische-Angiologische Praxis–Herzzentrum Bremen, Bremen, Germany; 3 Klinik für Kardiologie und Internistische Intensivmedizin, Johannes-Wesling-Klinikum Minden, Universitätsklinikum der Ruhr-Universität BochumHans-Nolte-Straße 1, Minden, Germany; University of Otago, NEW ZEALAND

## Abstract

**Introduction:**

Transradial coronary angiography (TRC) can be performed with a one-catheter approach for the right and left coronary ostium (R/LCO). We investigated the performance of a special diagnostic catheter widely used for the one-catheter-approach, the Tiger (Tiger II, Terumo^TM^).

**Methods:**

In a dual center registry we analyzed 1412 TRC-procedures exclusively performed by experienced TRC-operators. We compared the performance of the Tiger with Judkins catheters by retrospectively judging ostial catheter stability during contrast injection, and by measuring contrast use, fluoroscopy time (FT) and complication rate.

**Results:**

Poor or failed ostial engagement was found in 40.5% in the Tiger group, compared to 46.6% with the use of Judkins catheters (p<0.183). Ostial instability of the Tiger was found more often during engagement of the LCO than the RCO (34.4% vs. 10.8%, p<0.001), whereas it was similar in the LCO and RCO for Judkins catheters (27.4% vs. 26.7%, p = 0.840). TRC-procedures performed with Tiger catheters were associated with less contrast volume (63.48 ± 29.83mL vs. 82.51 ± 56.58mL, p<0.004) and shorter FT than with Judkins catheters. (198.27 ± 194.8sec vs. 326.85 ± 329.70sec). Forearm hematomas occurred less often with the Tiger (1.2% vs. 6.6%, p< 0.02).

**Conclusion:**

The Tiger employed as a single catheter in TRC is an effective tool to achieve lower contrast volume and fluoroscopy time at a low complication rate. Unstable engagement affects predominantly the left coronary artery, but its overall frequency is similar for both, the Tiger and Judkins catheters.

## Introduction

Transradial coronary angiography (TRC) and PCI were introduced by Campeau in 1989 [1] and Kiemeneij in 1992 [2]. The use of TRC has risen significantly due to its lower risk of bleeding and earlier postprocedural ambulation, especially for the high risk group of the elderly [3] [4] [5]. According to current guidelines it is the recommended access in STEMI [6, 7].

Difficult advancement of the catheter through tortuous and sometimes spastic arteries in the right arm and neck is an important disadvantage of TRC resulting in increased radiation exposure of the patient and the operator [8] [5] [9]. This led to the one-catheter-concept: a uniquely designed catheter which can be engaged in either coronary ostium [10–12]. It avoids any catheter exchange, thereby protecting upper limb vessels from repeated mechanical irritation and resulting in lower procedure and fluoroscopy times and lower cost. However, despite these advantages [11] many operators, including very experienced ones, reported difficulties maintaining a stable position within the coronary ostia during dye injection often resulting in disengagement of the catheter.

In the present registry of two centers favoring TRC, we investigated the one-catheter-concept of the widely used catheter “Tiger” (“Tiger II”; Terumo^TM^) [11, 13] by comparing catheter stability during contrast injection, contrast volume, fluoroscopy time (FT) and complication rate of the Tiger catheter to the Judkins catheters.

## Material and methods

This project has been registered and approved by the local ethics committees at the University Medical Centre Schleswig-Holstein in Kiel and at the Johannes-Wesling-Klinikum (University Medical Centre of the Ruhr-University Bochum) in Minden, Germany. All patients had given written informed consent prior to any data acquisition.

### Study design

In this dual centre registry we identified all diagnostic TRC procedures performed at the two participating catheterization laboratories in 2012 and 2013 [14]. All procedures were performed in a standard fashion from the right radial artery using 4F, 5F or 6F sheaths (Terumo^TM^). In both centers the Tiger is by far the most frequently used catheter for TRC (standard catheter). However, whether or not to use a Tiger or conventional catheters, was left to the discretion of the operator. For the one-catheter approach the Tiger 4 curve was primarily chosen in patients presenting with a body length of ≥175cm, 3.5 curve in case of <175cm. For the conventional approach Judkins left 3.5 and Judkins right 4 curves were used. Cases with acute coronary syndromes and cardiogenic shock were included. Excluded from the analysis were cases done by low volume operators, defined as < 80 TRC procedures per year, procedures with bypass graft angiography, primary left radial approach, procedures performed under the use of other diagnostic caths than the Tiger or Judkins catheters, and those with a planned or ad hoc PCI. In the exclusively evaluated diagnostic TRCs fluoroscopy time (taken during 8 cine angiography standard projections) and contrast volume (given by hand in all cases) were taken from catheterization reports. Charts and discharge summaries were searched for vascular complications.

Coronary angiograms (demonstrating stability of the catheter during contrast injection) were evaluated at both institutions by one and the same independent observer not involved in the procedure.

Stability was judged to be good if no disengagement was noted during injection of contrast into either left or right coronary artery (“0” was registered for stable). Poor ostial stability was defined as disengagement from the left coronary ostium (LCO) or the right coronary ostium (RCO) once or more than once during cinematography leading to semi-selective contrast injection and incomplete filling of the coronary artery (“1” was registered for unstable).

Failure of the Tiger catheter was defined as inability to achieve satisfactory catheter engagement from the right radial artery necessitating the use of a Judkins-type catheter or change of vascular access to the contralateral radial or femoral artery.

### Statistics

Statistical analysis was performed by the Institute of Medical Biometrics and Statistics of the Christian-Albrecht-University of Kiel, Germany applying the SPSS Statistics software (Version 21; SPSS Inc.; Chicago, Illinois). Continous data was expressed as mean value and standard deviation. All data was scanned whether normally distributed data. In case of not normal distribution the Wilcoxon signed rank test was performed. A two-tailed p value <0.05 was considered significant.

## Results

The entire study cohort from both centers consisted of n = 2953 consecutive TRC procedures. Exclusion according to the study criteria resulted in 1412 procedures included in the analysis. Trans-radial coronary angiography was performed with the Tiger as the primary catheter in 852 cases (%). Patient characteristics are shown in Table 1. Patients done with the Tiger catheter were slightly younger and were hypertensive more often than those examined with Judkins catheters. Overall, crossover from right to left radial access occurred in 5 cases (0.9%) and to femoral access 59 cases (2.0%). Left transradial procedures were excluded from analysis.

**Table 1:** Biometric data.

	Overall	Tiger II	Judkins	p-value Tiger II /Judkins
**Number of patients (n)**	1412	852	137	
**Age (years)**	66.78±11.96	65.74±12.19	70.17±12.46	<0.001
**Female (n; %)**	551 (39.02%)	366 (42.96%)	50 (36.50%)	0.163
**Body length (cm)**	172.51±9.42	171.76±9.49	172.50±9.59	0.468
**Body weight (kg)**	83.52±18.70	81.80±18.19	83.18±17.03	0.296
**BMI (kg/m**^**2**^**)**	27.95±5.40	27.60±5.23	27.81±5.14	0.756
**BSA (m**^**2**^**)**	1.96±0.23	1.94±0.23	1.95±0.22	0.502
**Diabetes mellitus (n; %)**	250 (17.71%)	149 (17.49%)	24 (17.52%)	>0.999
**HbA1c (%)**	5.95±0.77	5.93±0.82	5.95±0.61	0.378
**HLP (n; %)**	585 (41.43%)	347 (40.73%)	58 (42.34)	0.779
**LDL (mg/dl)**	114.10±38.84	116.82±40.10	111.03±38.24	0.319
**HDL (mg/dl)**	55.09±19.10	56.01±19.77	53.32±15.79	0.499
**Arterial hypertension (n; %)**	1029 (72.88)	616 (72.30)	91 (66.42)	<0.001
**Systolic blood pressure** **before angio (mmHg)**	136.81±28.00	138.95±27.86	130.20±28.36	0.003
**Diastolic blood pressure before angio (mmHg)**	73.36±15.17	73.83±15.25	73.39±15.19	0.757

The Tiger and Judkins do not add up to the overall column. This is due to the inclusion of 423 patients who were excluded when the Tiger was not successful.

BMI–Body mass index, BSA–Body surface area, HLP–Hyperlipoproteinemia

### Ostial stability (Fig 1 and Fig 2)

**Fig 1:**
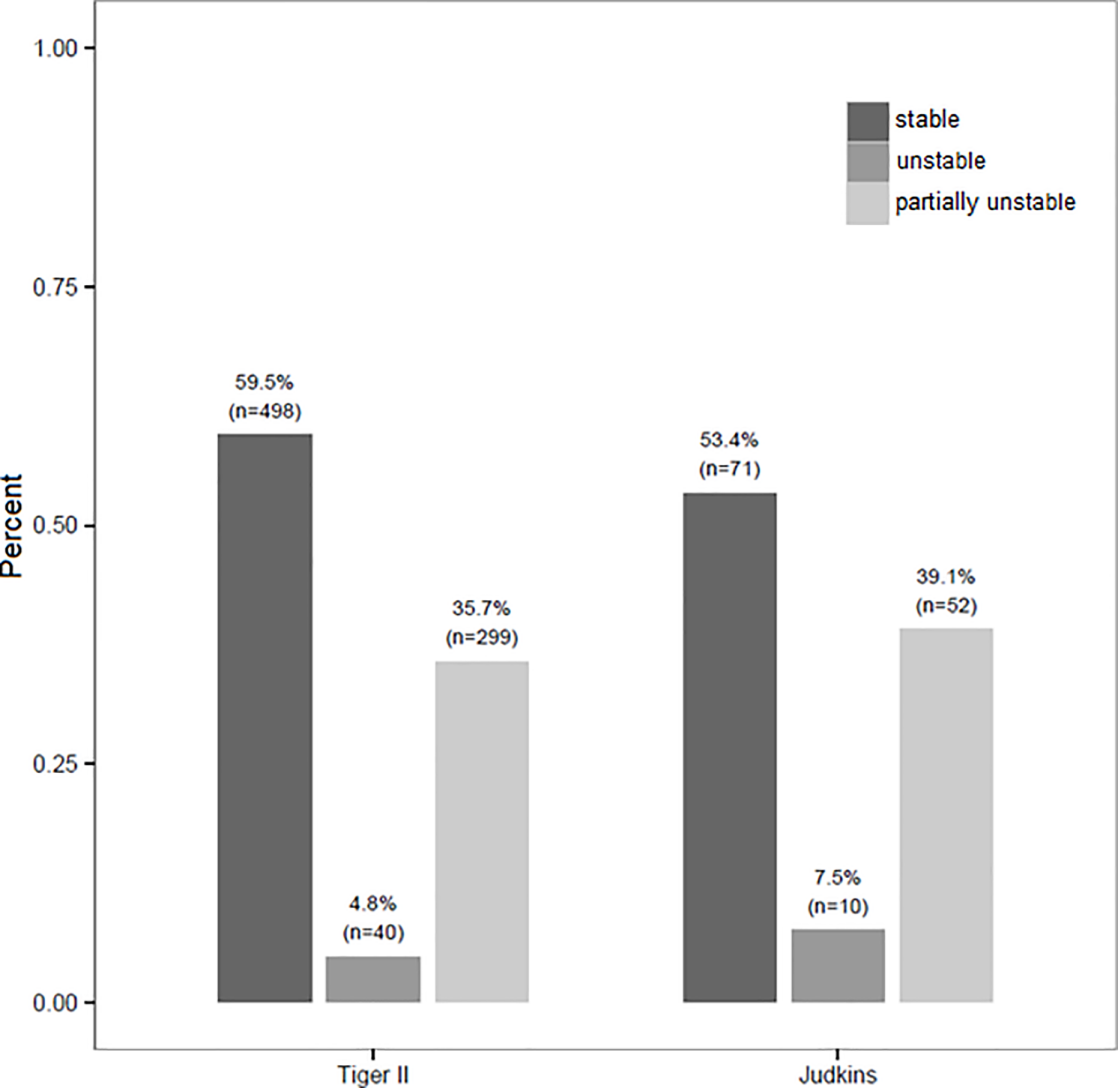
Ostial stability of the Tiger. The bar graph shows the distribution of stable (left), completely unstable (mid) and partially unstable (right) ostial catheter landing when using the Tiger and the Judkins catheters. “Stable” means stable ostial landing in both coronary ostia; “unstable” means instable ostial landing in both ostia. “Partially unstable” means unstable ostial landing in the RCO or LCO.

Ostial stability of the Tiger was deemed good in 498 cases (59.5%) and poor in 299 cases (35.7%). In 40 cases (4.8%), the Tiger failed completely to engage in either ostium. Poor ostial stability of the Tiger was seen more often in the LCO (290 cases; 34.0%) compared to the RCO (90 cases, 10.8%, p<0.001) (Fig 1).

**Fig 2:**
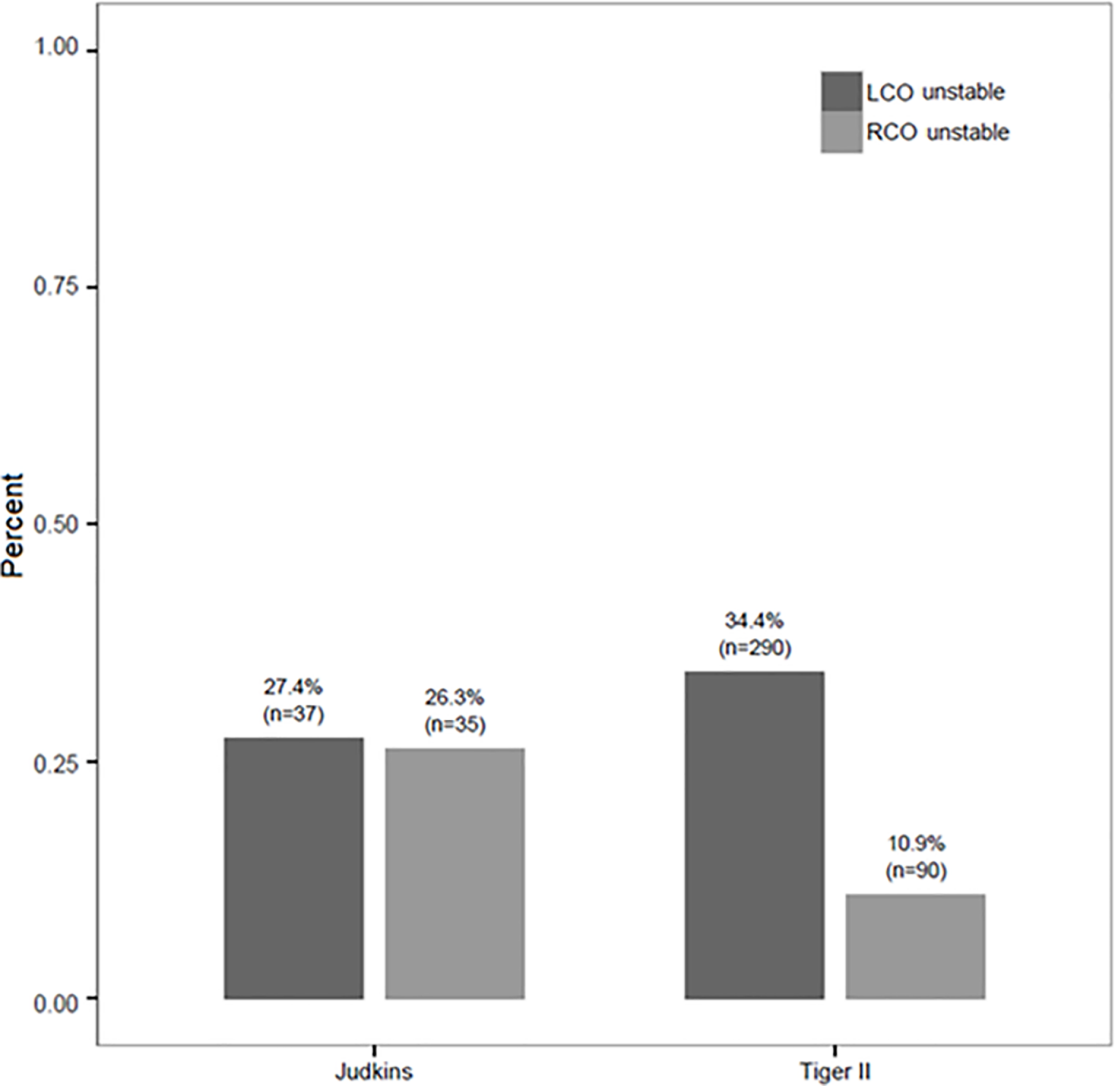
Distribution of ostial stability of the Tiger and Judkins catheters. The bar graph demonstrates the distribution of ostial instability within the RCO and LCO when using the Tiger and the Judkins catheters. Tiger vs. Judkins in the LCO p = 0.108, Tiger vs. Judkins in the RCO p<0.001, Judkins LCO vs. Judkins in the RCO p = 0.840, Tiger LCO vs. Tiger in the RCO p<0.001.

A switch to Judkins catheters due to poor or failed ostial engagement of Tiger occurred in 423 cases (33.2%). Compared to 137 TRC-procedures with Judkins catheters, no difference in the rate of poor ostial stability was found: 40.5% vs. 46.6% (p = 0.183).

Different from the Tiger, poor ostial stability of Judkins catheters was found at an equal rate in either coronary ostium (LCO 27.4% vs. RCO 26.3%). Poor ostial instability in the RCO occurred less often with the Tiger catheters compared to Judkins catheters (10.8% vs. 26.3%, p<0.001). In the left coronary ostium, Judkins catheters were found to have a lesser frequency of poor ostial engagement (34.4% vs. 27.4%, p = 0.108) (Fig 2).

### Contrast volume and fluoroscopy time

**Fig 3:**
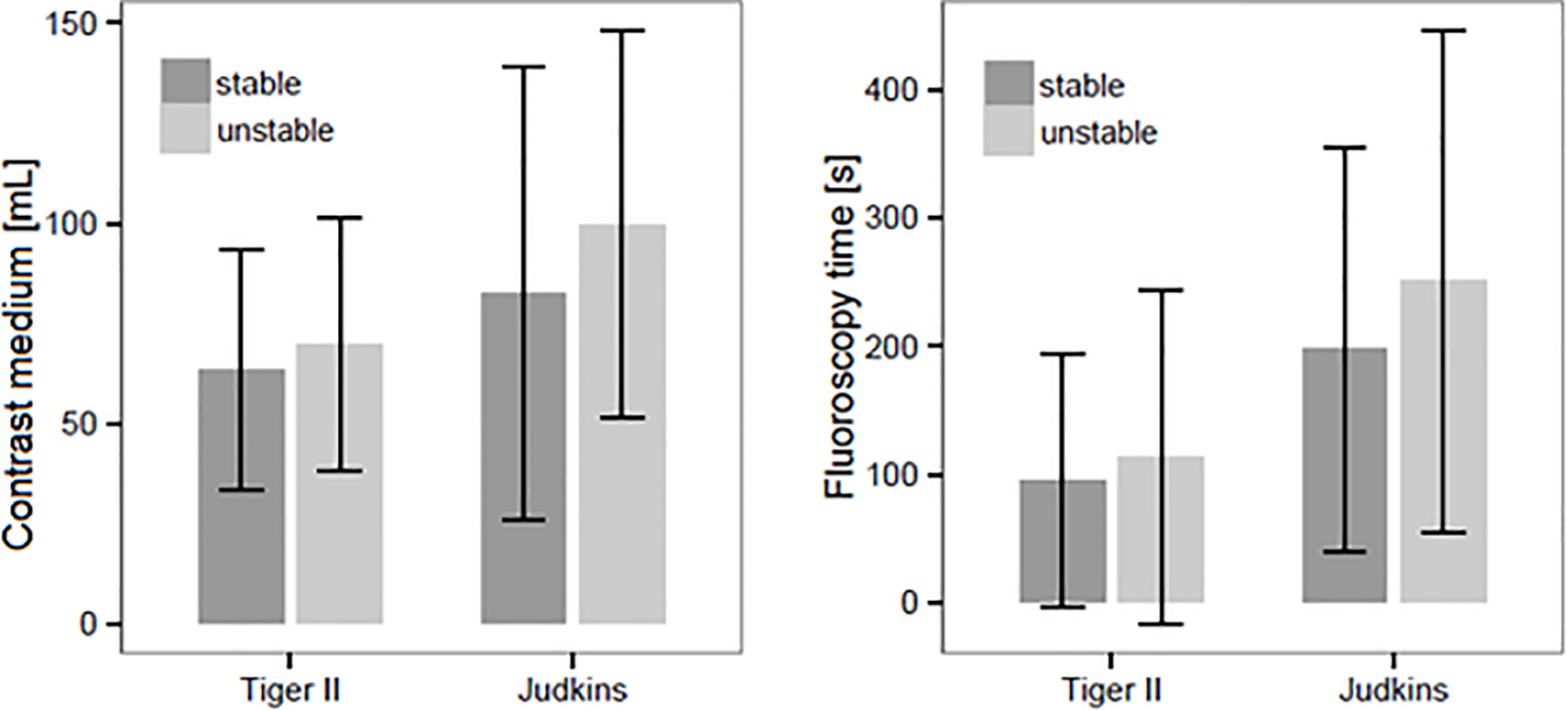
Dye volume and fluoroscopy time. The left bar graph demonstrates the volume of contrast medium applied by the Tiger and Judkins catheters in case of stable and unstable ostial landing (not normally distributed data); Tiger: p = 0.004, Judkins: p = 0.075. The right bar graph shows FT applied under the use of the Tiger and the Judkins in case of stable and instable ostial landing (not normally distributed data): Tiger p = 0.024, Judkins p = 0.255.

Procedures performed with a Tiger catheter were associated with significant less contrast volume compared to Judkins catheters (65.23 ± 30.69mL vs. 96.63 ± 55.27mL, p<0.001). This was the case whether or not difficulties with ostial engagement were encountered. Tiger use resulted in lower contrast volume than Judkins use in cases with good ostial stability (63.48 ± 29.83mL vs. 82 ± 56.58mL, p<0.004), as well as poor ostial stability (69.82 ± 31.76mL vs. 99.67±48.42mL, p<0.001). Likewise, FT was significantly lower for Tiger compared to Judkins catheters (198.27 ± 194.85 sec vs. 326.85 ± 329.7 sec, p<0.001), whether or not good ostial stability was present (Fig 3).

### Complication rate

Complications encountered consisted only of forearm hematomas associated with 5F and 6F sheaths; neither reported were documented spasms of the radial or brachial artery nor coronary complications. Complication rate of procedures performed with the Tiger catheter was lower than with Judkins catheters (1.6% vs. 6.6%, p<0.002). It increased slightly to 2.4% when a switch from Tiger to Judkins catheters was necessary.

## Discussion

The main reason for the development of a one-catheter-concept [4] [5] [15, 16] is the achievement of shorter fluoroscopy times, less contrast use and less radiation exposure. However, this goal can only be reached, if the Tiger catheter can deliver a stable engagement in both coronary ostia. The Tiger catheter has encountered fast growing acceptance, particularly in Asia, where it was reported to be the preferred catheter in two thirds of TRC’s. [13]. The performance of the Tiger has been investigated only by a small randomised single-centre study of 80 patients reported by Kim et al. who found successful coronary ostial engagement in 100% of the right and 91% of the left coronary artery [11]. However, among radialists, its efficacy has been in doubt, in particular its capability for stable seating in both coronary ostia. Consequently, we examined its efficacy in a large, retrospective multi-center cohort during routine trans-radial procedures.

Our study confirms that the Tiger catheter is a suitable tool for the implementation of the one-catheter-concept since it obviated the need for a second catheter in two thirds of the cases. However, poor ostial stability of the Tiger during contrast injection was seen in 35.7% of cases, of whom two thirds occurred in the left coronary ostium with the Tiger. The same frequency of ostial instability (33.2%) was encountered with the Judkins catheters which we attribute to the shapes of the Judkins curves which were designed for femoral access. Ostial instability in the left coronary artery was significantly more common in the Tiger than in the Judkins left curve, whereas it was significantly less common in the right coronary artery. Thus, poor performance of the Tiger curve predominantly affects the left coronary artery. For the right coronary artery, performance of the Tiger curve was significantly better than that of the Judkins right curve.

Not surprisingly, a negative impact of difficult ostial engagement on contrast volume and FT was confirmed by our results. However, this effect was less pronounced with the Tiger catheters, which showed this problem equally affects both curves. When we analyzed all cases regardless of the occurrence of ostial instability, contrast volume and FT were significantly less when a one-catheter approach was chosen. We believe the reason for this is the superior performance of the Tiger curve in engaging the right coronary ostium where most of the contrast volume and FT are spent.

Two previous studies investigating the one-catheter strategy reported contrast volumes during TRC with “Brachial Type K” and the Amplatzer Left (“AL”) procedure of 103 ± 33mL and 114 ± 78mL, respectively [17], which is considerably higher than the amount of the Tiger in our study (65 ± 31mL). There is no such data about the “Jacky”. However, a direct comparison to these earlier studies is not possible since coronary angiography in these catheterization laboratories may have been done in a different manor.

### Clinical implications

The non-inferiority of ostial stability of the Tiger, less contrast use and shorter FT, as well as lower complication rates are proof of its efficacy in day-to-day practice. Its mayor disadvantage is frequent ostial instability in the LCO leading to inadequate filling and switching to Judkins curves, which was seen in no less than 33% overall. Based on this observation, we advise operators to avoid using the Tiger catheter for diagnostic angiography in scenarios where a difficult engagement in the left coronary can be anticipated, such as aortic ectasia of aortic valve stenosis. Despite this draw-back, contrast use and FT were lower than with the two-catheter approach with Judkins curves which we believe is due to the vastly superior performance of the Tiger reaching the RCO. This has implications for patients with renal failure or poor left ventricular function, in whom contrast use and FT are crucial. In these groups, we propose choosing the one-catheter-approach with the Tiger or a hybrid approach with Tiger for the right and a Judkins left catheter for the left coronary artery if Tiger fails to engage promptly. Further prospective studies are needed to prove this approach to be superior in high risk patients. Finally, we observed a lower incidence of bleeding complications in the group where the operator intended to use only the Tiger catheter. Again, we believe that the Tiger one-catheter approach should be the first choice in patients at high risk of bleeding complications, such as elderly females.

### Limitations

Due to its retrospective study design, catheter performance was measured by judging ostial stability during contrast injection. This surrogate parameter may only partially reflect ease of engagement in the coronary ostia. Furthermore, we did not record separately FT for cannulation of each coronary ostium, nor do we have data on the frequency of switching to another catheter size or an Amplatz curve. The retrospective nature of our study did not exclude bias of the operator in his choice of the catheter. Patients in the Judkins group were older than in the Tiger group possibly reflecting a bias to use conventional catheters for patients deemed have a more complicated catheterization procedure.

## Supporting information

S1 Data Excel-database. (XLSX)

## References

[1] Campeau L. Percutaneous radial artery approach for coronary angiography. Cathet Cardiovasc Diagn. 1989;16(1):3–7. Epub 1989/01/01. . 2912567 10.1002/ccd.1810160103

[2] Kiemeneij F, Laarman GJ. Percutaneous transradial artery approach for coronary Palmaz-Schatz stent implantation. Am Heart J. 1994;128(1):167–74. Epub 1994/07/01. . 8017270 10.1016/0002-8703(94)90023-x

[3] Hamon M, Pristipino C, Di Mario C, Nolan J, Ludwig J, Tubaro M, et al Consensus document on the radial approach in percutaneous cardiovascular interventions: position paper by the European Association of Percutaneous Cardiovascular Interventions and Working Groups on Acute Cardiac Care** and Thrombosis of the European Society of Cardiology. EuroIntervention. 2013;8(11):1242–51. Epub 2013/01/29. doi: 10.4244/EIJV8I11A192 20130115–02 [pii]. . 23354100 10.4244/EIJV8I11A192

[4] Alnasser SM, Bagai A, Jolly SS, Cantor WJ, Dehghani P, Rao SV, et al Transradial approach for coronary angiography and intervention in the elderly: A meta-analysis of 777,841 patients. Int J Cardiol. 2017;228:45–51. Epub 2016/11/20. doi: S0167-5273(16)33684-1 [pii] doi: 10.1016/j.ijcard.2016.11.207 . 27863361 10.1016/j.ijcard.2016.11.207

[5] Sciahbasi A, Frigoli E, Sarandrea A, Rothenbuhler M, Calabro P, Lupi A, et al Radiation Exposure and Vascular Access in Acute Coronary Syndromes: The RAD-Matrix Trial. J Am Coll Cardiol. 2017;69(20):2530–7. Epub 2017/03/24. doi: S0735-1097(17)36042-4 [pii] doi: 10.1016/j.jacc.2017.03.018 . 28330794 10.1016/j.jacc.2017.03.018

[6] Ibanez B, James S, Agewall S, Antunes MJ, Bucciarelli-Ducci C, Bueno H, et al 2017 ESC Guidelines for the management of acute myocardial infarction in patients presenting with ST-segment elevation: The Task Force for the management of acute myocardial infarction in patients presenting with ST-segment elevation of the European Society of Cardiology (ESC). Eur Heart J. 2017 Epub 2017/09/10. doi: 10.1093/eurheartj/ehx393 4095042 [pii]. .28886621

[7] Windecker S, Kolh P, Alfonso F, Collet JP, Cremer J, Falk V, et al 2014 ESC/EACTS Guidelines on myocardial revascularization: The Task Force on Myocardial Revascularization of the European Society of Cardiology (ESC) and the European Association for Cardio-Thoracic Surgery (EACTS)Developed with the special contribution of the European Association of Percutaneous Cardiovascular Interventions (EAPCI). Eur Heart J. 2014;35(37):2541–619. Epub 2014/09/01. doi: 10.1093/eurheartj/ehu278 ehu278 [pii]. . 25173339 10.1093/eurheartj/ehu278

[8] Lange HW, von Boetticher H. Randomized comparison of operator radiation exposure during coronary angiography and intervention by radial or femoral approach. Catheter Cardiovasc Interv. 2006;67(1):12–6. Epub 2005/12/07. doi: 10.1002/ccd.20451 . 16331696 10.1002/ccd.20451

[9] Rigattieri S, Valsecchi O, Sciahbasi A, Tomassini F, Limbruno U, Marchese A, et al Current practice of transradial approach for coronary procedures: A survey by the Italian Society of Interventional Cardiology (SICI-GISE) and the Italian Radial Club. Cardiovasc Revasc Med. 2017. Epub 2017/01/26. doi: S1553-8389(17)30007-6 [pii] doi: 10.1016/j.carrev.2017.01.005 . 28119043 10.1016/j.carrev.2017.01.005

[10] Ikari Y, Ochiai M, Hangaishi M, Ohno M, Taguchi J, Hara K, et al Novel guide catheter for left coronary intervention via a right upper limb approach. Cathet Cardiovasc Diagn. 1998;44(2):244–7. Epub 1998/06/24. doi: 10.1002/(SICI)1097-0304(199806)44:2<244::AID-CCD24>3.0.CO;2-L [pii]. . 9637453 10.1002/(sici)1097-0304(199806)44:2<244::aid-ccd24>3.0.co;2-l

[11] Kim SM, Kim DK, Kim DI, Kim DS, Joo SJ, Lee JW. Novel diagnostic catheter specifically designed for both coronary arteries via the right transradial approach. A prospective, randomized trial of Tiger II vs. Judkins catheters. Int J Cardiovasc Imaging. 2006;22(3–4):295–303. Epub 2005/12/06. doi: 10.1007/s10554-005-9029-8 . 16328852 10.1007/s10554-005-9029-8

[12] Zhang Q, Qiu JP, Zhang RY, Hu J, Yang ZK, Ding FH, et al Improved outcomes from transradial over transfemoral access in primary percutaneous coronary intervention for patients with acute ST-segment elevation myocardial infarction and upstream use of tirofiban. Chin Med J (Engl). 2013;126(6):1063–8. Epub 2013/03/20. . 23506579

[13] Bertrand OF, Rao SV, Pancholy S, Jolly SS, Rodes-Cabau J, Larose E, et al Transradial approach for coronary angiography and interventions: results of the first international transradial practice survey. JACC Cardiovasc Interv. 2010;3(10):1022–31. Epub 2010/10/23. doi: 10.1016/j.jcin.2010.07.013 S1936-8798(10)00557-1 [pii]. . 20965460 10.1016/j.jcin.2010.07.013

[14] Langer C, Riehle J, Frey N, Wiemer M. Catheter stability in transradial coronary angiography: The one-catheter-concept and the impact of performance level in 1,419 patients. Int J Cardiol. 2015;187:680–2. Epub 2015/04/18. doi: S0167-5273(15)00562-8 [pii] doi: 10.1016/j.ijcard.2015.03.322 . 25885590 10.1016/j.ijcard.2015.03.322

[15] Jolly SS, Amlani S, Hamon M, Yusuf S, Mehta SR. Radial versus femoral access for coronary angiography or intervention and the impact on major bleeding and ischemic events: a systematic review and meta-analysis of randomized trials. Am Heart J. 2009;157(1):132–40. Epub 2008/12/17. doi: 10.1016/j.ahj.2008.08.023 S0002-8703(08)00742-4 [pii]. . 19081409 10.1016/j.ahj.2008.08.023

[16] Sciahbasi A, Romagnoli E, Trani C, Burzotta F, Sarandrea A, Summaria F, et al Operator radiation exposure during percutaneous coronary procedures through the left or right radial approach: the TALENT dosimetric substudy. Circ Cardiovasc Interv. 2011;4(3):226–31. Epub 2011/05/19. doi: 10.1161/CIRCINTERVENTIONS.111.961185 CIRCINTERVENTIONS.111.961185 [pii]. . 21586692 10.1161/CIRCINTERVENTIONS.111.961185

[17] Sanmartin M, Esparza J, Moxica J, Baz JA, Iniguez-Romo A. Safety and efficacy of a multipurpose coronary angiography strategy using the transradial technique. J Invasive Cardiol. 2005;17(11):594–7. Epub 2005/11/03. . 16264204

